# CEST MRI assessment of HIV-1-associated neurometabolic impairments in a humanized mouse model

**DOI:** 10.1515/nipt-2025-0017

**Published:** 2025-12-29

**Authors:** Gabriel C. Gauthier, Micah Summerlin, Balasrinivasa R. Sajja, Mariano G. Uberti, Emma G. Foster, Manjeet Kumar, Matthew Thiele, Santhi Gorantla, Aditya N. Bade, Yutong Liu

**Affiliations:** Department of Radiology, University of Nebraska Medical Center, Omaha, NE, USA; Department of Pharmacology and Experimental Neuroscience, University of Nebraska Medical Center, Omaha, NE, USA; Department of Radiology, University of Nebraska Medical Center, Omaha, NE, USA; Department of Radiology, University of Nebraska Medical Center, Omaha, NE, USA; Department of Pharmacology and Experimental Neuroscience, University of Nebraska Medical Center, Omaha, NE, USA; Department of Pharmacology and Experimental Neuroscience, University of Nebraska Medical Center, Omaha, NE, USA; Department of Pharmacology and Experimental Neuroscience, University of Nebraska Medical Center, Omaha, NE, USA; Department of Pharmacology and Experimental Neuroscience, University of Nebraska Medical Center, Omaha, NE, USA; Department of Pharmacology and Experimental Neuroscience, University of Nebraska Medical Center, Omaha, NE, USA; Department of Radiology, University of Nebraska Medical Center, Omaha, NE, 68198, USA; and Department of Pharmacology and Experimental Neuroscience, University of Nebraska Medical Center, Omaha, NE, USA

**Keywords:** human immunodeficiency virus (HIV), antiretroviral therapy (ART), HIV-associated neurocognitive disorders (HAND), magnetic resonance imaging (MRI), chemical exchange saturation transfer (CEST), humanized mice

## Abstract

**Objectives:**

Human immunodeficiency virus 1 (HIV-1)-associated neurocognitive disorders (HAND) persist despite antiretroviral therapy (ART), driven by ongoing neuroinflammation and metabolic dysfunction. This study assesses whether chemical exchange saturation transfer (CEST) MRI can detect HIV-1–induced neurometabolic impairments and ART-mediated improvements in a humanized mouse model.

**Methods:**

HIV-1–infected CD34-NSG mice underwent CEST MRI at baseline (Week 0), 6 weeks post-infection (6 WPI), and after 6 weeks of ART or vehicle treatment (12 WPI). CEST contrast was quantified at 2 ppm (creatine-related), 3 ppm (glutamate-related), and −3.5 ppm (nuclear Overhauser effect, NOE). Neuroinflammation and infection were evaluated using immunohistochemistry and qPCR.

**Results:**

At 6 WPI, HIV-1 infection reduced 2-ppm CEST contrast in the cortex and hippocampus and increased NOE in the cortex. By 12 WPI, vehicle-treated mice showed decreased 3-ppm contrast in the cortex, hippocampus, and piriform cortex, whereas ART restored contrast in the cortex and hippocampus. Vehicle-treated mice also showed reduced 2-ppm contrast in the cortex, hippocampus, piriform cortex, and thalamus; ART restored this in the hippocampus, piriform cortex, and thalamus. Increased NOE at −3.5 ppm was observed but did not show measurable improvement following ART. CEST alterations corresponded with decreased HIV-1 p24+ cells and reduced neuroinflammatory markers in ART-treated brains.

**Conclusions:**

CEST MRI detects region-specific metabolic abnormalities during HIV-1 infection and region-specific metabolic recovery with ART, consistent with reduced viral burden and neuroinflammation. These findings support CEST MRI as a promising non-invasive biomarker for monitoring treatment response and disease progression in neuroHIV.

## Introduction

Although antiretroviral therapy (ART) has transformed human immunodeficiency virus type 1 (HIV-1) infection into a manageable chronic illness, treatment outcomes remain highly variable, and important clinical challenges remain [1–6]. Effective viral suppression requires lifelong adherence, which can be challenging due to personal, social, and financial barriers [1–6]. Even with consistent access to care and undetectable viral loads, a significant proportion of people living with HIV-1 (PLWH) continue to experience HIV-1-associated neurocognitive disorders (HAND). HAND encompasses a spectrum of neurocognitive impairments, including asymptomatic neurocognitive impairment (ANI), mild neurocognitive disorder (MND), and HIV-1-associated dementia (HAD) [7–15]. Any of these impairments can adversely affect daily functioning and quality of life [7–16].

HAND is closely linked to persistent neuroinflammation and neuroimmune dysfunction driven by long-lived viral reservoirs within the central nervous system (CNS), particularly in microglia and perivascular macrophages [7–16]. The blood-brain barrier (BBB) restricts ART penetration into the CNS [17–22], resulting in suboptimal drug concentrations and facilitating the persistence of low-level viral replication [7, 14, 23–26]. Sustained viral and neuroimmune activity contributes to synaptic injury, glial activation, and chronic metabolic disturbances that persist despite ART [7, 8, 12–14]. Furthermore, several antiretrovirals (ARVs) have been implicated in mitochondrial stress, impaired glial function, and neuronal toxicity [2, 4–10, 12–14, 16].

Beyond neuroimmune injury, numerous studies have documented metabolic abnormalities in the HIV-affected brain. MRS and metabolomic analyses have shown reduced glutamate levels in cortical and subcortical regions [27, 28], impaired astrocytic glutamate uptake, and disruptions in the glutamate–glutamine cycle in HIV-exposed glia (e.g., EAAT2 downregulation) [29–31]. Similarly, bioenergetic disturbances including altered creatine and phosphocreatine levels [32, 33], changes in lactate-related pathways [34], and disrupted astrocyte–neuron energy coupling [35, 36], have been reported in PLWH. These metabolic impairments reflect early glial stress, excitatory neurotransmission dysregulation, and mitochondrial dysfunction; all of which are increasingly recognized as contributors to HAND pathology.

Although the neuroimmune mechanisms of HAND are well characterized, fewer studies have evaluated how HIV-1 infection and ART dynamically alter CNS metabolites *in vivo*, particularly in small-animal models. Given the strong metabolic component of neuroHIV, sensitive molecular imaging tools capable of detecting neurometabolic shifts may help address this gap. This study employs a chemical exchange saturation transfer (CEST) magnetic resonance imaging (MRI) approach to characterize HIV-1–associated neurometabolic alterations and ART responses in a humanized mouse model. CEST MRI is an advanced molecular imaging technique that enables non-invasive detection of biomolecules, even at low concentrations, through proton exchange processes [16, 37–46]. By applying a frequency-selective saturation to exchangeable solute protons, CEST MRI detects attenuation of the water signal due to proton transfer, generating contrasts sensitive to specific solutes. CEST contrasts used in the study correspond to 3 ppm that is associated with amine proton exchange in glutamate [41, 47–53]; 2 ppm that is creatine-related guanidinium proton exchange [54–59], and −3.5 ppm resulting from relayed nuclear Overhauser effect (NOE) of macromolecules (e.g., mobile proteins, lipids) [60–62]. Because metabolic abnormalities are strongly implicated in HAND, CEST MRI may provide sensitive imaging biomarkers complementary to traditional MRS and neuropathology.

Here, we apply CEST MRI to a humanized mouse model of HIV-1_ADA_ infection to assess longitudinal neurometabolic alterations and evaluate metabolic restoration following ART. By integrating CEST findings with virologic, immunologic, and qualitative neuropathological assessments, this study aims to determine whether CEST MRI can serve as a non-invasive imaging biomarker for HAND-related metabolic disruption and ART-mediated recovery.

## Materials and methods

### Generation of humanized mice and HIV-1 infection

All animal studies included in this manuscript were approved by the University of Nebraska Medical Center (UNMC) Institutional Animal Care and Use Committee (IACUC) per the standards of the Guide for the Care and Use of Laboratory Animals (National Research Council of the National Academies, 2011). Male CD34-NSG humanized mice (n = 14) were used to evaluate the effects of HIV-1_ADA_ infection and ART on CNS metabolic profiles. These mice are widely used to mimic human immune responses and study HIV-1-linked neuropathology [63, 64]. Detailed procedures for generating humanized mice are described in previous studies [63]. Briefly, NSG (NOD.Cg-Prkdc^scid^Il2rgt^m1Wjl^/SzJ) mice were bred and maintained at UNMC under pathogen-free conditions per the ethical guidelines set forth by the National Institutes of Health (NIH) for the care of laboratory animals. Human CD34+ hematopoietic stem progenitor cells (HSPCs) were isolated from human cord blood obtained from healthy full-term newborns (Department of Gynecology and Obstetrics, UNMC). CD34+ cells were isolated using immune-magnetic beads (CD34+ selection kit; Miltenyi Biotec Inc., Gaithersburg, MD). The purity (> 90 %) and number of CD34+ cells were confirmed using flow cytometry. On post-natal day 1–3, neonates received 1 Gy irradiation using an RS-2000 X-ray Irradiator (Rad Source Technologies) for 4 h, and were intrahepatically (i.h.) injected with 10^5^ CD34+ HSPCs in 20 μL phosphate-buffered saline (PBS) using a 30-gauge needle. Human cell engraftment was confirmed by flow cytometry of peripheral blood collected by cheek-bleeding. At 20–22 weeks of age, mice with >20 % human CD45+ cells were selected for the study. HIV-1 infection was initiated by intraperitoneal (i.p.) injection of HIV-1_ADA_ at 10^4^ tissue culture infectious dose_50_ (TCID_50_).

### Experimental design

**Figure 1:**
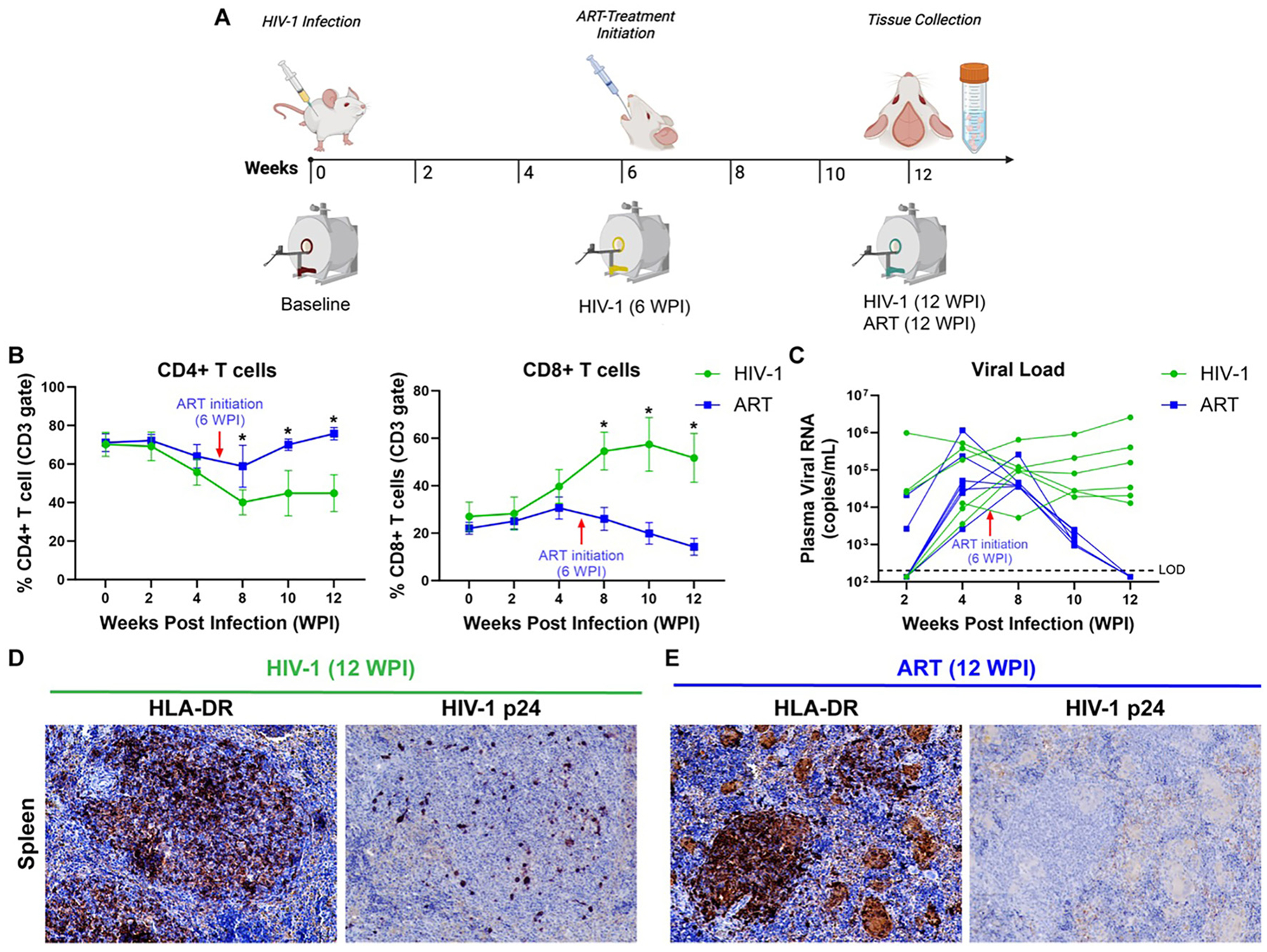
Experimental timeline of HIV-1 infection and antiretroviral therapy in humanized mice. (A) Experimental design: Humanized mice underwent MRI scans before HIV-1 infection at baseline (0 WPI), followed by biweekly cheek bleeding starting at 2 WPI to assess plasma viral load and T cell counts. A second MRI was conducted at 6 WPI, before initiating daily ART treatment, with a final MRI scan at 12 WPI. Euthanasia was conducted following MRI for tissue collection. (B) Flow cytometry results: CD4+ and CD8+ T cell counts were tracked post-infection and following ART or vehicle treatment. (C) Plasma viral RNA: Dynamics of plasma viral load post-infection and after treatment were measured using the automated COBAS ampliprep system. Immunohistological analysis of spleen at 12 WPI in (D) vehicle- and (E) ART-treated mice.

Experimental design is illustrated in Figure 1A. Baseline imaging, including T_2_-weighted anatomical scans and CEST MRI, was conducted on humanized mice before HIV-1 infection (Week 0). Following the baseline scan, the mice were infected with HIV-1 as described above. At 6 weeks post-infection (WPI), infection was confirmed in all animals by measuring plasma viral load. Immediately after viral confirmation, animals were scanned again to determine the short-term effects of infection. After the 6 WPI scan, HIV-1-infected mice were randomly divided into two treatment groups: ART (n = 7) and vehicle (Control; n = 7). Animals were treated daily with ART or vehicle for 6 weeks, from 6 WPI to 12 WPI. Imaging was repeated once more at 12 WPI to assess the effects of long-term infection and ART treatment. Some mice were removed from the study after exhibiting predefined signs of graft-versus-host disease, including weight loss, skin changes such as erythema or lesions, hunched posture, lethargy, or ruffled fur. Five vehicle-treated mice and four ART-treated mice successfully underwent 12 WPI MRI scans. Mice were euthanized immediately after final imaging. Brain and spleen tissues were collected for immunohistological analyses. Peripheral blood was collected biweekly from the submandibular vein (cheek bleed) to measure temporal plasma viral RNA and CD4+/CD8+ T-cell counts as biomarkers of HIV-1 infection (Figure 1A). Blood was not collected at 6 WPI to avoid additional stress during combined MRI scan and ART initiation.

### ART administration

HIV-1 infected animals were administered ART or vehicle daily by oral gavage, from 6 WPI to 12 WPI (Figure 1A). The ART regimen was comprised of tenofovir disoproxil (123 mg/kg; mouse weight), lamivudine (123 mg/kg; mouse weight), and dolutegravir (20.5 mg/kg; mouse weight) in combination, also known as TLD. The dosage corresponds to double the human equivalent dose (HED) of TLD [65, 66]. TLD was selected because it is widely used in resource-limited settings and provides potent viral suppression across diverse HIV-1 subtypes [67–69]. The supratherapeutic dosage ensured that the plasma viral load reached undetectable levels within 6–8 weeks, given the rapid rise in plasma viral load (exceeding 10^6^ copies/mL within 4 weeks) observed in this high-titer infection model. The vehicle [dimethylsulfoxide:Solutol^®^:50 mM N-methylglucamine in 3 % mannitol (1:1:8, v:w:v)] [70], utilized to create a suspension of the drugs, was administered to controls at the same volume and scheme as the TLD administration.

### Measurements of plasma viral load and T cell counts

Mouse peripheral blood samples were taken biweekly from the submandibular vein (cheek bleed) by using 5 mm lancets (MEDIpoint, Inc., Mineola, NY) and collected in EDTA-coated tubes. Quantitative viral load measurements were conducted using the automated COBAS Ampliprep system v2.0/Taqman-48 system (Roche Molecular Diagnostics, Basel, Switzerland) as described previously [71–73]. The limit of detection for viral load measurement is 200 viral RNA copies/mL according to the dilution factor [71]. In parallel, the peripheral blood human-immune-cell profile was examined by flow cytometry. After the collection of excess plasma, 25 μL of whole blood suspensions were incubated with a combination of human monoclonal antibodies for CD45, CD3, CD4, and CD8 markers for 30 min [63, 71, 73]. Red blood cells (RBCs) were lysed with FACS lysing solution (BD Biosciences, Franklin Lakes, NJ, USA), and finally stained cells were fixed with 2% paraformaldehyde (PFA). Flow cytometry was carried out with a standard procedure using the LSR-II FACS analyzer [63]. Targeted-cell-specific populations were analyzed using FlowJo v10.5 (BD Pharmingen, San Diego, CA, USA), and percentages of the total number of gated lymphocytes were expressed as results.

### Magnetic resonance imaging (MRI)

MRI was performed on a 7T Bruker PharmaScan system using a Bruker quadrature mouse head coil. Mice were anesthetized with 1–3% isoflurane, and respiration and was monitored throughout scanning. Body temperature was maintained at 37 °C using a water-heating bed throughout imaging.

### T_2_-weighted MRI

A RARE sequence was used to acquire T_2_-weighted images on axial planes with the following parameters: TE = 48 ms, TR = 4.2 s, 4 averages, RARE factor = 8, number of slices = 21, slice thickness = 0.5 mm, FOV = 20 × 20 mm, and matrix = 256 × 192.

### CEST MRI

Prior to all CEST scans, first- and second-order B0 shimming was performed over the brain volume using the vendor-provided field-map–based shimming routine to ensure optimal field homogeneity. CEST MRI data were acquired using a RARE sequence with a saturation RF amplitude of 2 μT, a duration of 2 s, and a frequency offset range from −5 to 5 ppm. An offset step = 0.2 ppm was used in the ranges of [−5 ppm, −4 ppm] and [4 ppm, 5 ppm], while an offset step = 0.1 ppm was used in [−4 ppm, 4 ppm]; this non-uniform frequency sampling allowed us to adequately observe all relevant offsets, while prioritizing accuracy within the central range where CEST contrasts at 2 and 3 ppm and NOE at −3.5 ppm are observed. The scan included two slices that were positioned to include cortex, hippocampus, hypothalamus, piriform cortex, and thalamus, with FOV = 20 × 20 mm^2^, slice thickness = 0.5 mm, and matrix = 256 × 256. The main magnetic field (B0) correction was performed using WASSR [74].

Preprocessing steps included motion inspection, rigid-body coregistration of all offset images to the corresponding T_2_-weighted anatomical image, and voxelwise alignment across frequency offsets prior to Lorentzian fitting. All analyses, including ROI placement and curve fitting, were performed blinded to treatment group. CEST data was analyzed using five-pool Lorentzian fitting for offsets at 2 ppm, 3 ppm, and −3.5 ppm, −1 ppm, and 0 ppm. The CEST contrasts at 2 and 3 ppm are associated with creatine and glutamate, respectively. The contrast at −3.5 ppm is associated with NOE of macromolecules, and the contrast at −1 ppm results from the magnetization transfer (MT) from semi-solid macromolecules. Direct water suppression is fitted at 0 ppm. CEST contrast was quantified as the area under each fitted Lorentzian curve, integrated over a width defined as 5% of the peak amplitude. The 5% Lorentzian width threshold was selected because it provides a to balance between estimation robustness and sensitivity to contrast variations, consistent with prior small animal CEST studies [75, 76]. The accuracy of Lorentzian fitting was examined through both residual plots and goodness-of-fit evaluations (Supplementary Figures 2–3 and Supplementary Table 1). Specifically, the coefficient of determination (R^2^) was computed for each pixel, and only those with R^2^ ≥ 0.8 were included in the region-of-interest (ROI) analyses. Representative R^2^ maps and residuals are presented in Supplementary Figure 2, and group-wise summary statistics are provided in Supplementary Table 1. ROIs (cortex, hippocampus, thalamus, hypothalamus, piriform cortex) were manually defined on CEST maps using T_2_-weighted images as an anatomical reference to extract region-specific values.

### Immunohistology

At 12 WPI, mice were humanely euthanized immediately after imaging, and brains and spleens were collected. Tissues were fixed in 4% PFA overnight. Later fixed tissues were processed in the Epredia^™^ STP 120 Spin Tissue Processor (Epredia^™^, Thermo Fisher Scientific, Waltham, MA, USA) using the standard overnight protocol, followed by embedding in paraffin blocks. Tissue sections of 5 μm in thickness were collected and stained with mouse monoclonal antibodies for HLA-DQ/DR/DP (Novus Biologicals, LLC, Centennial,), or HIV-1 p24 (Santa Cruz Biotechnology, Inc., Dallas, TX), and rabbit polyclonal antibodies for glial fibrillary acidic protein (GFAP) (Dako, Carpinteria, CA), or ionized calcium binding adaptor molecule −1 (Iba-1) (FUJIFILM Wako Pure Chemical Corporation, Osaka, Japan). The polymer-based HRP-conjugated anti-mouse and anti-rabbit Dako EnVision systems were used as secondary detection reagents. 3,3′-diaminbenzidine (DAB, Dako, Carpinteria, CA) was used as a chromogen. All paraffin-embedded sections were counterstained with Mayer’s hematoxylin. Images were captured with a 20 × objective using the Nuance EX multispectral imaging system (CRi, Wobum, MA, USA) [63].

### RT-qPCR assay

Total RNA was isolated from brain cortex tissues using TRIzol reagent (Invitrogen, Waltham, MA, USA). The RNA concentration was measured using a nanodrop spectrophotometer (Thermo Fisher Scientific, Waltham, MA, USA). Further, cDNAs were synthesized from RNA using Verso cDNA synthesis kits (Thermo Fisher Scientific), as per the manufacturer’s protocol. The synthesized cDNA was used as a template for reverse transcription-quantitative polymerase chain reaction (RT-qPCR). Real-time PCR amplification was performed on the Quant Studio^™^ 5 System using SYBR green master mix (Applied Biosystems, Waltham, MA, USA). The HIV-1 RNAs were quantified using the following primers. For HIV-1 Gag, FP 5′-ATCTGGCCTGGTGCAATAGG-3 and RP 5′-ACATCAAGCAGCCATGCAAAAT-3; for GAPDH, FP 5- TGAGCAAGAGAGGCCCTATC-3 and RP 5-AGGCCCCTCCTGTTATTATG-3. The RT-qPCR was performed under the following conditions: 50 °C for 2 min, 95 °C for 10 min, 45 cycles of 95 °C for 15 s and 60 °C for 1 min. The fold change of the target gene (HIV-1 Gag) expression was calculated using the ΔΔC_T_ method after normalization with an endogenous mouse GAPDH transcript expression of total RNA.

### Statistical analysis

All analyses were conducted using GraphPad Prism. Normality of the data was assessed within each brain region using quantile–quantile (Q–Q) plots (Supplementary Figure 3). Post-hoc comparisons between baseline and 6 WPI data were evaluated using two-tailed Student’s t-tests to assess the effects of early infection. Comparisons between baseline and 12 WPI data from vehicle-treated HIV-infected mice (to assess prolonged infection effects) used Welch’s corrected two-tailed t-tests to account for unequal variance and sample size. To assess the effects of ART, comparisons between ART-treated and vehicle-treated HIV-infected mice at 12 WPI were also conducted using Welch’s two-tailed t-tests. Bonferroni correction was applied when appropriate to adjust for multiple comparisons. Although longitudinal designs often use mixed-effects modeling, the small sample size and attrition at 12 WPI limited the feasibility of a full repeated-measures framework. Therefore, t-tests were used to evaluate specific time-point contrasts appropriate for this exploratory study. A simple mixed-effects model was additionally evaluated to confirm that major conclusions were consistent across statistical approaches. Statistical significance was set at p < 0.05, and data are expressed as mean±standard error of the mean (SEM), with a minimum of three biological replicates. Symbols used for significance: #p < 0.1, *p < 0.05, **p < 0.01, ***p < 0.001, ****p < 0.0001.

## Results

### Viral and immune profile of HIV-1 infected humanized mice

Productive infection was confirmed by plasma viral load and immune parameters through 12 WPI (Figure 1B and C). Flow cytometry was used to track temporal changes in CD4+ and CD8+ T cells in both treatment groups (in Figure 1B and Supplementary Figure 1). The steady decline in CD4+ T cells was consistently observed in all mice following HIV-1 infection. Levels of CD4+ T cells declined from 70% at baseline to 56% by 4 WPI in mice from the vehicle-treated group (green color) and from 71% at baseline to 64 % by 4 WPI in mice from the ART-treated group (blue color). After the initiation of treatment at 6 WPI, in the vehicle-treated mice, CD4+ cells continued to decline, reaching 45 % at 12 WPI. In contrast, CD4+ cells in ART-treated mice increased from 64 % at 4 WPI to 76 % at 12 WPI. During the treatment period, CD4+ T cells were significantly higher in ART-treated mice compared to vehicle-treated mice at 8 WPI (p = 0.009), 10 WPI (p = 0.04), and 12 WPI (p = 0.02). Parallel to CD4+ T declines, CD8+ T cells increased following infection (Figure 1B). Levels of CD8+ T cells increased from 27 to 40 % (vehicle) and 22–31 % (ART) by 4 WPI. After treatment initiation, CD8+ T cells continued to rise in vehicle-treated mice (reaching 52 % at 12 WPI) but declined in ART-treated mice from 31 % at 4 WPI to 14 % at 12 WPI. ART-treated mice displayed significantly lower CD8+ percentages than vehicle-treated mice at 8, 10, and 12 WPI (p = 0.01, 0.02, 0.01). To confirm productive viral infection, plasma viral RNA levels were quantified (Figure 1C). Viral loads peaked at ~4 WPI in all mice. In vehicle-treated mice, plasma viral loads ranged from 3.56 × 10^3^ to 5.23 × 10^5^ RNA copies/mL, while ART-treated mice ranged from 2.60 × 10^3^ to 1.16 × 10^6^ RNA copies/mL prior to treatment. After six weeks of treatment, viral loads remained high in vehicle-treated mice (1.29 × 10^4^ to 2.57 × 10^6^) but fell below the limit of detection (LOD) in all ART-treated mice, confirming treatment efficacy (Figure 1C).

Immunohistology further verified human immune cell reconstitution and HIV-1 infection in spleens (Figure 1D and E). At 12 WPI, robust HLA-DR staining confirmed human immune cell distribution in both groups, including white pulp, red pulp, and germinal centers (Figure 1D and E). HIV-1 p24 staining showed numerous infected cells in vehicle-treated mice but no detectable p24+ cells in ART-treated mice (Figure 1D and E), confirming sustained infection in vehicle-treated mice and markedly reduced viral burden in ART-treated mice.

### HIV-1-associated changes in brain contrast at 3 ppm and ART-mediated recovery

**Figure 2:**
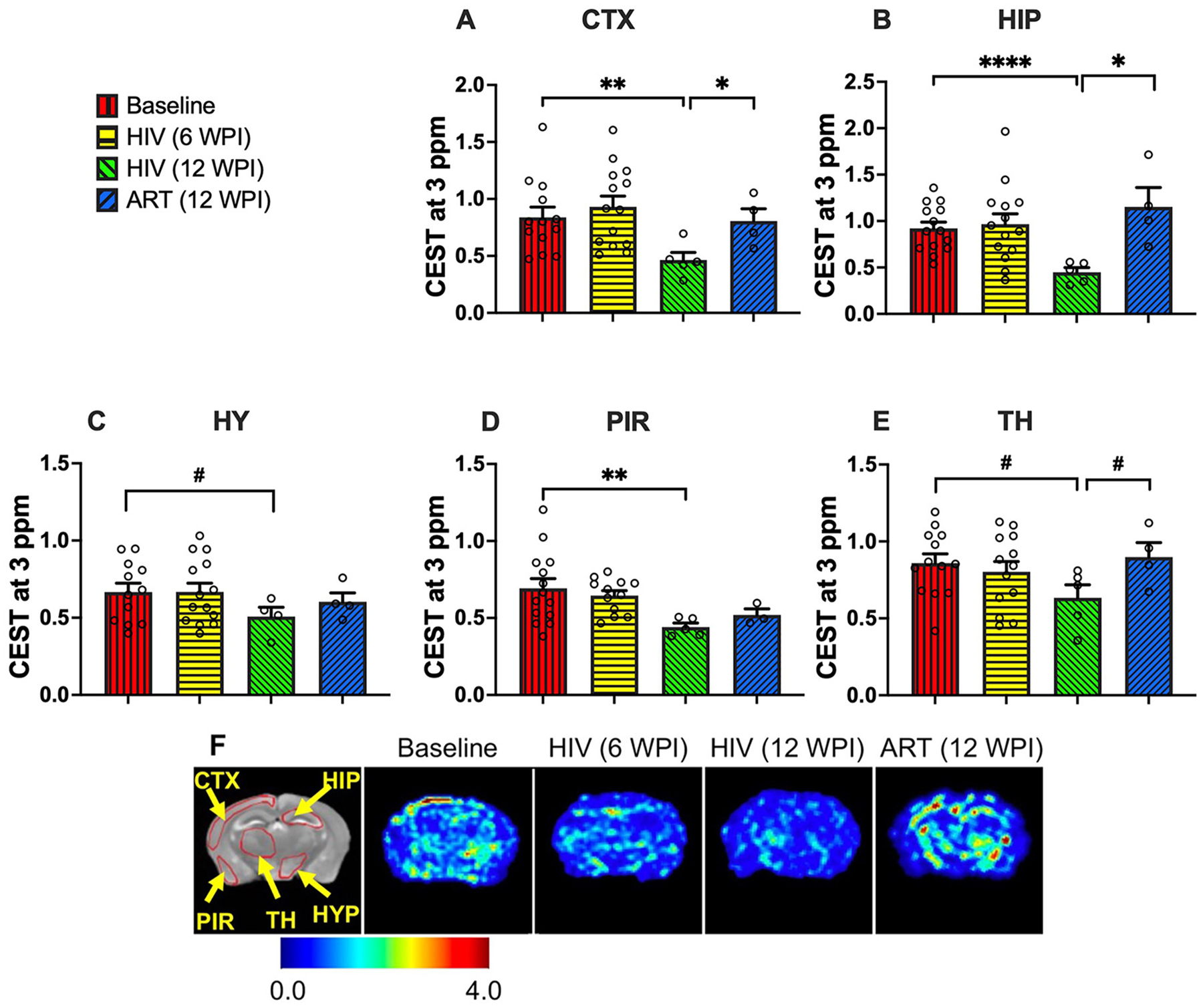
CEST contrast values at 3 ppm (associated with glutamate) in (A) cortex (CTX), (B) hippocampus (HIP), (C) hypothalamus (HY), (D) piriform cortex (PIR), and (E) thalamus (TH) regions. The experimental groups shown are baseline (red bars), HIV-infected at 6 WPI (yellow bars), HIV-infected at 12 WPI (green bars), and ART-treated at 12 WPI (blue bars). #: 0.05 <. p < 0.1, *: 0.01 < p < 0.05, **: 0.001 < p < 0.01, ***: 0.0001 < p < 0.001, ****p < 0.0001. (F) 3 ppm contrast heatmaps of a representative mouse at baseline, a representative HIV-infected mouse at 6 WPI, a representative HIV-infected mouse at 12 WPI, and a representative ART-treated mouse at 12 WPI.

CEST contrast at 3 ppm is largely associated with glutamate [41, 49, 77]. Thus, to determine the adverse effect of HIV-1 infection on glutamate levels and the benefits of ART treatment for reversing these changes, CEST contrast at 3 ppm was quantified in five brain regions (Figure 2A–F): cortex, hippocampus, hypothalamus, piriform cortex, and thalamus. At 6 WPI, no region showed significant differences compared to baseline (Week 0), indicating that the short duration of productive infection (6 weeks) did not yet alter glutamate-weighted CEST signals. However, at 12 WPI, vehicle-treated mice exhibited significant reductions in 3 ppm contrast in the cortex (p = 0.004), hippocampus (p < 0.0001), and piriform cortex (p = 0.002), with trends toward reduction in the hypothalamus (p = 0.09) and thalamus (p = 0.06). These results suggest reduced glutamate-associated contrast after prolonged infection. Interestingly, ART-treated mice displayed significantly higher CEST contrast at 3 ppm at 12 WPI in the cortex (p = 0.05) and hippocampus (p = 0.04) compared to vehicle-treated mice, with a similar trend in the thalamus (p = 0.06). Importantly, 3 ppm contrast in ART-treated mice was comparable to baseline, indicating metabolic recovery.

Heatmaps of 3 ppm contrast on representative mouse brains are shown in Figure 2F. A T_2_-weighted image shows the anatomical references for the brain regions utilized to quantify 3 ppm contrasts. In the representative heatmaps, decreased intensity was clearly visible in vehicle-treated mice at 12 WPI compared to baseline, supporting reduced glutamate-associated contrast after prolonged infection. In contrast, ART-treated mice displayed higher 3 ppm contrast compared to vehicle-treated, with intensities similar to baseline, indicating recovery of glutamate-associated metabolic signal following ART. These data underscore the benefits of ART treatment in restoring 3 ppm contrast in HIV-1-infected mice.

### HIV-1 -induced alterations in the contrast at 2 ppm and ART-mediated restoration

**Figure 3:**
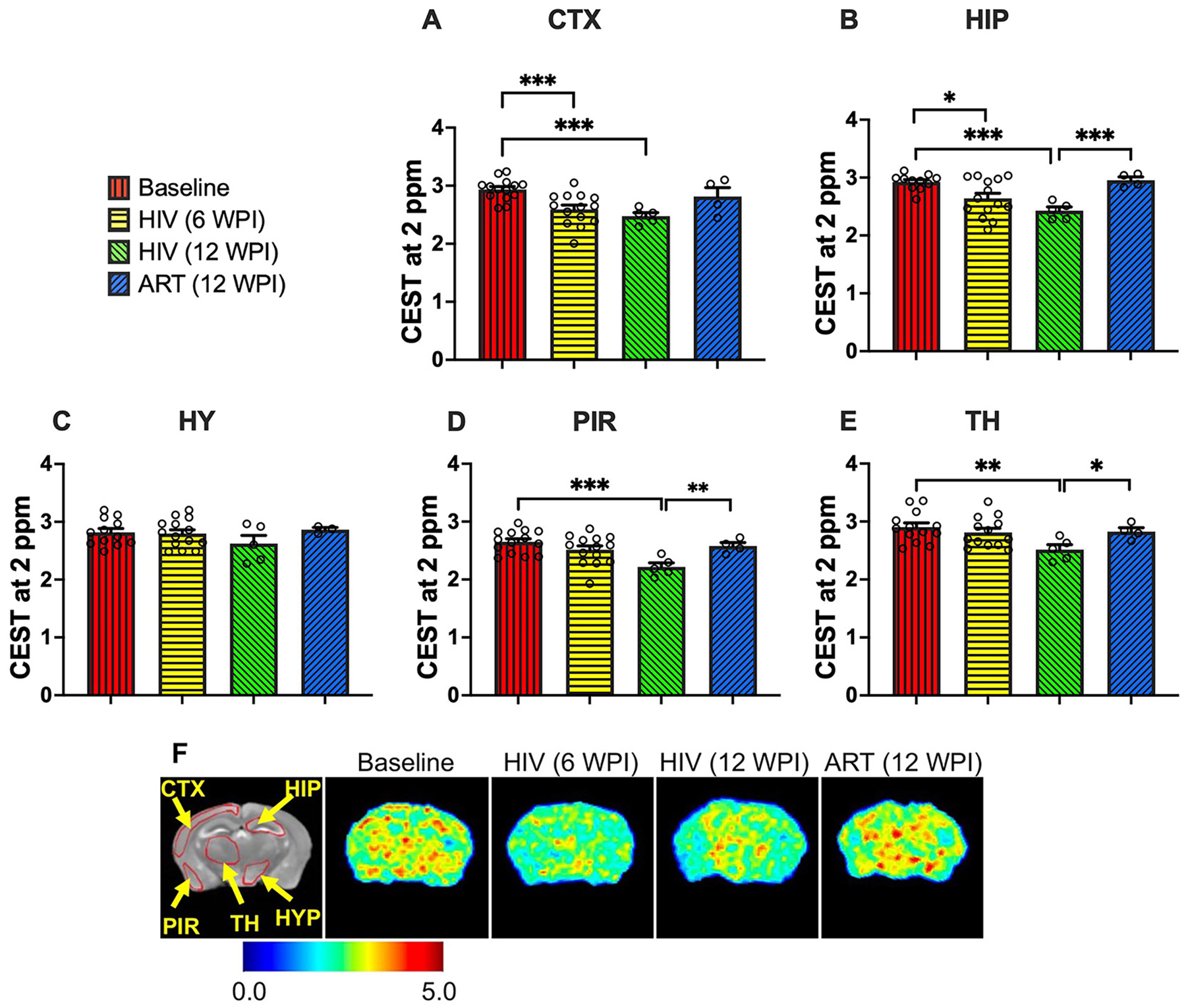
CEST contrast values at 2 ppm (associated with creatine) in (A) cortex (CTX), (B) hippocampus (HIP), (C) hypothalamus (HY), (D) piriform cortex (PIR), and (E) thalamus (TH) regions. The experimental groups shown are baseline (red bars), HIV-infected at 6 WPI (yellow bars), HIV-infected at 12 WPI (green bars), and ART-treated at 12 WPI (blue bars). #: 0.05 < p < 0.1, *: 0.01 < p < 0.05, **: 0.001 < p < 0.01, ***: 0.0001 < p < 0.001, ****p < 0.0001. (F) 2 ppm contrast heatmaps of a representative mouse at baseline, a representative HIV-infected mouse at 6 WPI, a representative HIV-infected mouse at 12 WPI, and a representative ART-treated mouse at 12 WPI.

CEST contrasts at 2 ppm are linked to creatine levels [44, 55, 78, 79]. Therefore, to identify the effects of HIV-1 on creatine levels and whether ART treatment aids in their recovery, CEST contrast at 2 ppm was measured in the same five brain regions as 3 ppm measurements (Figure 3A–F). At 6 WPI, the cortex (p = 0.0006) and hippocampus (p = 0.01) showed significantly reduced 2 ppm contrast compared to baseline, indicating that short-term (6 weeks) HIV-1 infection adversely affects creatine-associated contrast. At 12 WPI, vehicle-treated mice exhibited significant reductions in 2 ppm CEST contrast compared to baseline in the cortex (p = 0.0002), hippocampus (p = 0.0003), piriform cortex (p = 0.0009), and thalamus (p = 0.006). These reductions suggest a progressive decline in creatine-associated contrast with prolonged infection. In contrast, ART-treated mice demonstrated significantly elevated 2 ppm CEST contrast, compared to vehicle-treated mice at 12 WPI, in the hippocampus (p = 0.0005), piriform cortex (p = 0.0062), and thalamus (p = 0.028). In addition, 2 ppm CEST contrast in ART-treated mice closely matched baseline levels, indicating recovery of creatine-associated contrast.

Heatmaps of 2 ppm contrast from representative mouse brains are shown in Figure 3F. Heatmap patterns reflect the quantitative findings reported above. In vehicle-treated mice, reduced color intensity at 6 WPI and at 12 WPI was observed relative to baseline, consistent with declines in creatine-associated contrast. In ART-treated mice, higher color intensity was observed compared to vehicle, closely matching baseline values. These findings highlight the beneficial effect of ART on restoring 2 ppm CEST contrast after HIV-1–induced metabolic impairment.

### HIV-1-linked changes in NOE at −3.5 ppm

**Figure 4:**
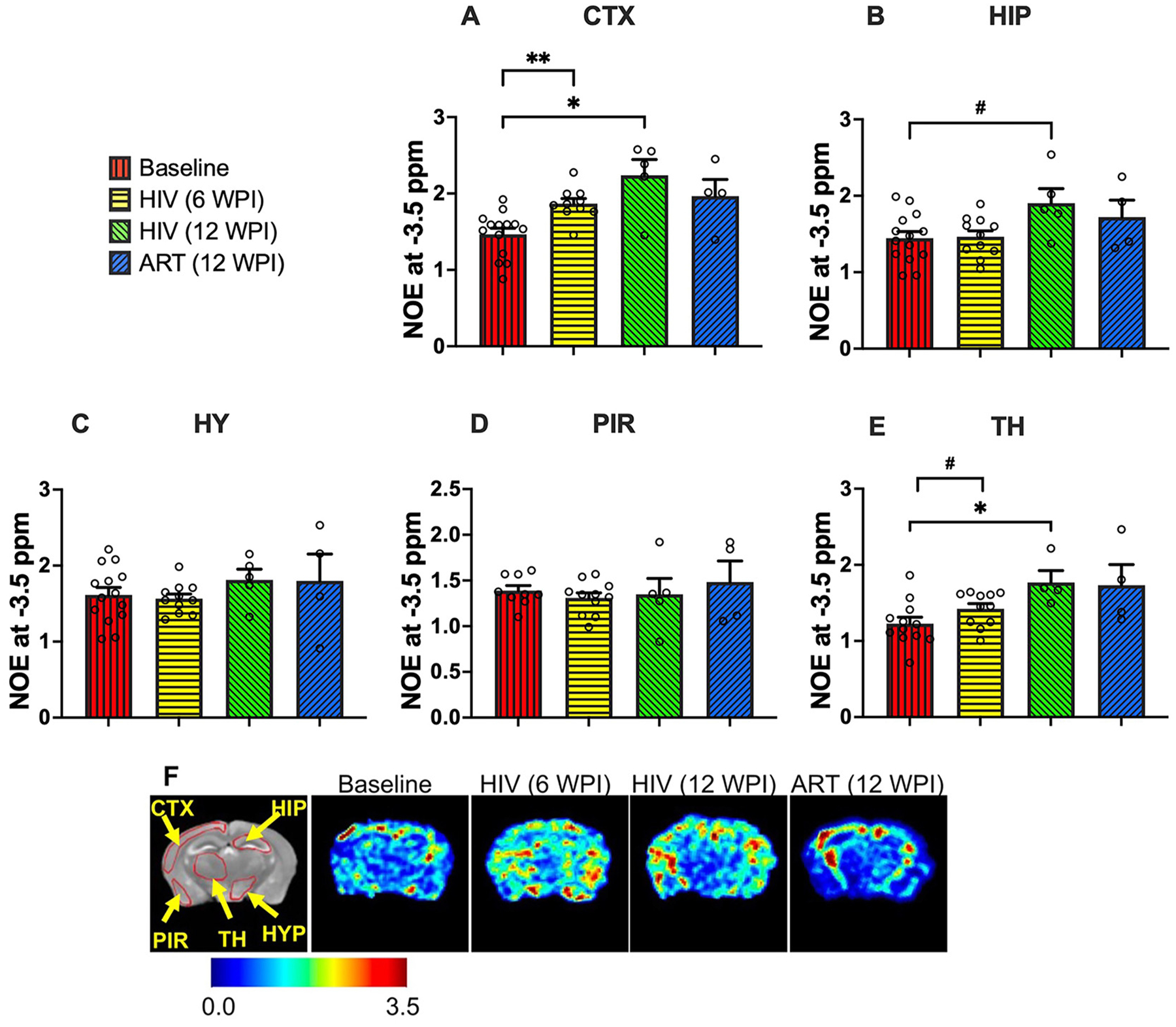
NOE contrast values (associated with membrane lipids) in (A) cortex (CTX), (B) hippocampus (HIP), (C) hypothalamus (HYP), (D) piriform cortex (PIR), and (E) thalamus (TH) regions. The experimental groups shown are baseline (red bars), HIV-infected at 6 WPI (yellow bars), HIV-infected at 12 WPI (green bars), and ART-treated at 12 WPI (blue bars). #: 0.05 < p < 0.1, *: 0.01 < p < 0.05, **: 0.001 < p < 0.01, ***: 0.0001 < p < 0.001, ****p < 0.0001. (F) NOE contrast heatmaps of a representative mouse at baseline, a representative HIV-infected mouse at 6 WPI, a representative HIV-infected mouse at 12 WPI, and a representative ART-treated mouse at 12 WPI.

NOE at −3.5 ppm is associated with exchange processes from brain macromolecules (e.g., mobile proteins, lipids) [60–62]. To examine HIV-1-induced macromolecular changes, NOE at −3.5 ppm was quantified across the same five brain regions (Figure 4A–F). At 6 WPI, the cortex showed a significant increase in NOE (p = 0.001), and the thalamus exhibited a trend toward elevation (p = 0.09) compared to baseline, reflecting early macromolecular or membrane-associated alterations during HIV-1 infection. By 12 WPI, vehicle-treated mice demonstrated continued elevation of NOE, with significant increases in the cortex (p = 0.02) and thalamus (p = 0.03), and a trend in the hippocampus (p = 0.07), relative to baseline. These findings suggest that macromolecular changes persist and may progress with prolonged infection, consistent with chronic neuroinflammation. In contrast to the 2 and 3 ppm contrasts, no significant differences were observed between ART- and vehicle-treated mice at 12 WPI in any region examined. These results indicate that ART did not produce measurable reductions in NOE within the study timeframe, suggesting that macromolecular abnormalities may be less responsive, or slower to respond, to ART than creatine- and glutamate-associated changes. Heatmaps of NOE from representative mouse brains are shown in Figure 4F. Compared to baseline, higher color intensity was observed at 6 WPI, reflecting early HIV-1–associated increases in NOE. At 12 WPI, vehicle-treated mice exhibited further increases, consistent with quantitative findings, whereas ART-treated mice showed elevated NOE similar to vehicle-treated mice, indicating a lack of detectable ART-mediated recovery. Together, these data suggest that NOE-associated macromolecular or membrane-related changes arise early after HIV-1 infection and persist despite ART.

### HIV-1 infection in mouse brains

**Figure 5:**
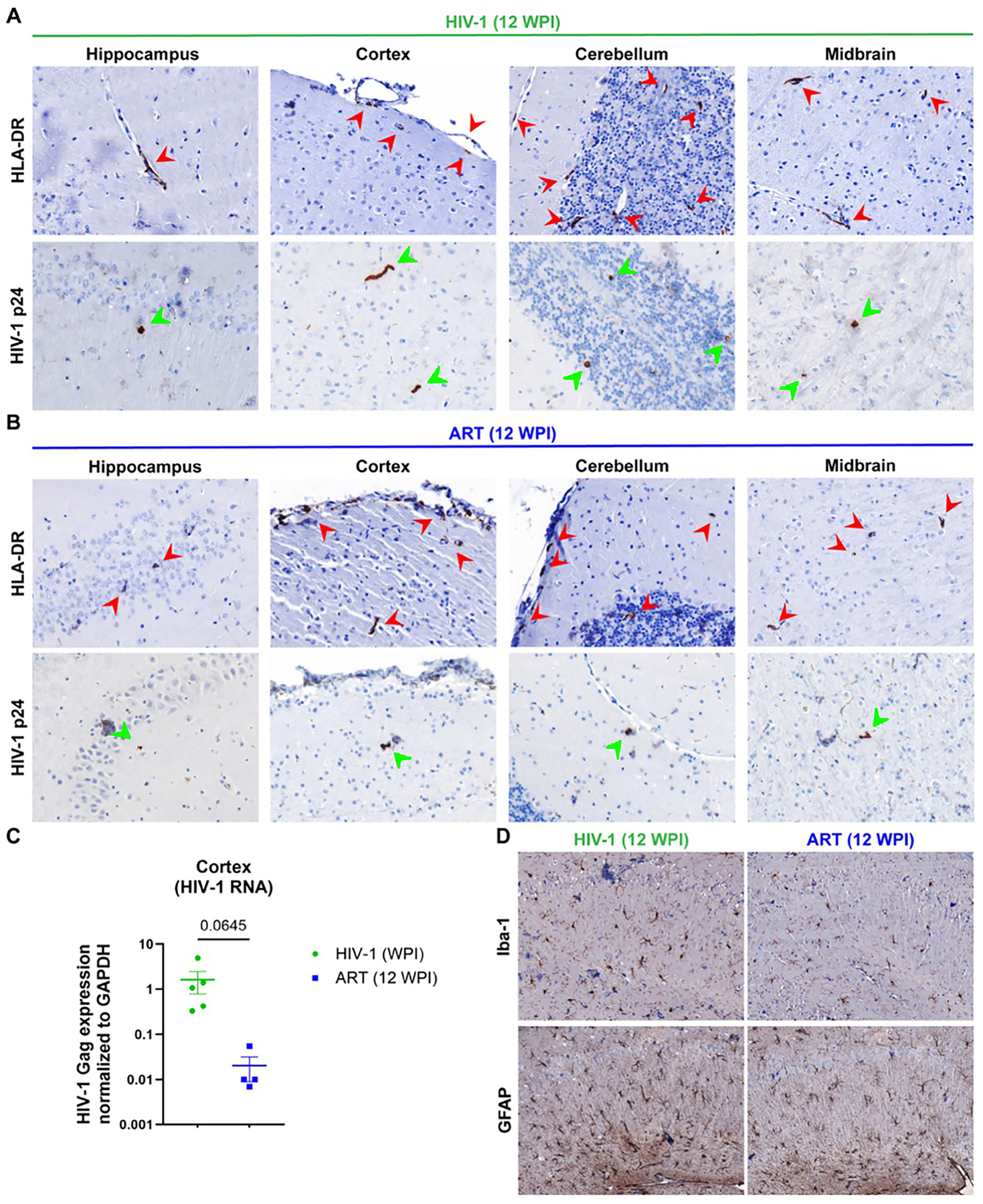
Immunohistochemical (IHC) analysis of brain regions from vehicle- and ART-treated HIV-infected humanized mouse models at 12 WPI. IHC staining for HLA-DR and HIV-1 p24 in the hippocampus, cortex, cerebellum, and midbrain from (A) vehicle-treated and (B) ART-treated HIV-1-infected mice. Red (HLA-DR) and green (HIV-1 p24) arrowheads indicate areas of positive staining. (C) HIV-1 Gag expression in the cortex of vehicle-treated (green) and ART-treated (blue) mice. (D) Comparative IHC staining for Iba-1 (microglia marker) and GFAP (astrocyte marker) in vehicle- and ART-treated mouse brain hippocampal sections.

To investigate brain infiltration of human cells, immunohistochemistry was performed at study termination (12 WPI). Brain sections were stained for human HLA-DR and HIV-1 p24 antigens. Human HLA-DR+ cells were detected within the brains of both vehicle- and ART-treated mice, appearing in multiple regions including the hippocampus, cortex, cerebellum, and midbrain (Figure 5A and B; indicated by red arrows, top row). HIV-1 p24+ human cells were observed in the same regions in vehicle-treated mice (Figure 5A and B; indicated by green arrows, bottom row). While total human immune-cell infiltration was similar between groups, the number of HIV-1 p24+ cells was markedly lower in ART-treated mice, reflecting reduced viral burden. Viral infection in the cortex was determined by real-time qPCR targeting HIV-1-Gag to quantify tissue viral RNA (Figure 5C). Vehicle-treated mice showed high cortical viral RNA levels, validating sustained viral infection, whereas ART-treated mice showed substantially lower viral RNA, consistent with effective viral suppression. Though viral infection was undetectable in plasma and spleen of ART-treated animals at 12 WPI, low-level of HIV-1 RNA was detectable in brain tissue. Both immunohistology and qPCR tests confirmed effective ART-mediated reduction, but not complete elimination of brain viral infection.

Further, glial cell responses were assessed by glial fibrillary acidic protein (GFAP, astrocyte) and ionized calcium binding adaptor molecule-1 (Iba-1, microglia) staining (Figure 5D). In vehicle-treated mice, robust glial activation was observed, including hypertrophic astrocytes, and enlarged microglial cell bodies. These morphological features are characteristic of virus-induced reactive gliosis. ART-treated mice showed reduced glial activation, consistent with decreased brain viral burden. However, a smaller population of astrocytes and microglia still exhibited activated morphologies, indicating residual neuroinflammation despite ART. Overall, glial staining patterns demonstrate that ART substantially reduces, but does not fully normalize, HIV-1–associated neuroinflammation in humanized mouse brains.

## Discussion

This exploratory study demonstrates significant neurometabolic perturbations in a humanized mouse model of HIV-1 infection. Notably, the study highlights the persistent adverse effects of infection on the neuroimmune system despite the potent efficacy of ART in controlling viral replication at undetectable levels. The application of CEST MRI in this preliminary feasibility framework provides insights into HIV-1-associated metabolic impairments and into the extent to which these abnormalities may improve with ART. These findings underscore the potential role of advanced neuroimaging techniques in unraveling HAND pathophysiology and in monitoring therapeutic outcomes.

Numerous previous studies have linked the contrasts at 3 and 2 ppm with glutamate and creatine [41, 44, 49, 50, 54–57, 59, 78, 79]. In this study, reductions in these contrasts in HIV-1–infected mice offer strong evidence of HIV-1-induced neurometabolic disruptions and support the use of CEST MRI for detecting treatment effects. In vehicle-treated HIV-1-infected mice, significant reductions at both contrasts suggest decreases in glutamate- and creatine-associated signals. Glutamate is a primary excitatory neuro-transmitter, and reductions in glutamate levels are indicative of glutamatergic dysfunction and neurodegenerative processes associated with chronic HIV-1 infection [7, 27, 28, 31]. In addition, alterations in 2 ppm CEST contrast likely reflect creatine metabolism, which is associated with cellular bioenergetic distress caused by HIV-1 infection [64, 80, 81]. These findings align with prior MRS and cellular studies in PLWH showing reduced glutamate [27, 28], altered creatine/energy homeostasis [32, 33], impaired astrocytic glutamate uptake [29–31], and disrupted astrocyte–neuron metabolic coupling [35, 36]. Together, these findings reinforce that metabolic injury is a central component of neuroHIV.

The distinct temporal patterns of creatine- and glutamate-associated signal alterations provide new insight into the neurobiological trajectory of HIV-1 neuropathology (Supplementary Figure 4). The early reduction in creatine-associated contrast observed at 6 WPI may signify an initial phase of astrocytic bioenergetic stress. Creatine serves as a vital component of the cellular energy buffering system via the phosphocreatine shuttle, particularly within astrocytes, which support neuronal energy metabolism. HIV-induced glial activation and mitochondrial dysfunction can compromise this metabolic equilibrium, resulting in diminished creatine availability or utilization. Over time, this energy imbalance may disrupt astrocyte-mediated glutamate uptake and recycling, eventually leading to excitotoxicity and progressive neuronal injury. The later reductions in glutamate-associated contrast at 12 WPI may therefore reflect downstream glutamatergic vulnerability secondary to sustained metabolic and inflammatory stress. Because these mechanistic interpretations cannot be directly validated without molecular assays, we frame them conservatively as potential explanations that are consistent with prior literature. Future studies integrating molecular markers of energy metabolism and neuroinflammation will be crucial to validating this framework.

The observed recovery of glutamate- and creatine-associated contrasts following ART highlights its neuroprotective potential. Furthermore, flow cytometry and plasma viral RNA evaluations confirmed the ART-linked decrease in HIV-1 infection in the peripheral system and CNS. ART treatment led to recovery of contrasts at 3 and 2 ppm. However, this recovery should be interpreted as partial normalization relative to untreated infected controls rather than as complete metabolic restoration. Previous human studies show that metabolic abnormalities can persist – and correlate with neurocognitive impairment – even in PWH on suppressive ART, and our findings are broadly consistent with that literature.

Changes of NOE at −3.5 ppm revealed an HIV-1-linked increase in macromolecular content, with no detectable reversal by ART within the study period. Previous studies suggest that NOE arises from macromolecular content and possibly membrane lipids [62]. The limited duration of ART treatment may partly explain the lack of recovery, as macromolecular remodeling and lipid turnover generally occur more slowly than metabolic restoration. This preliminary observation is consistent with changing neuroinflammation (micro- and astrogliosis), which was mitigated in the ART-treated group but not completely resolved. Importantly, NOE elevations require cautious interpretation. Increased NOE may reflect a range of underlying changes, including altered lipid composition, protein conformation, water-macromolecule exchange dynamics, or even local magnetic field inhomogeneities, and therefore does not definitely indicate neuronal injury.

Metabolic impairments were observed across multiple brain regions, with broadly distributed patterns. Infiltration of human HIV-1 p24+ cells confirmed widespread CNS involvement. This broad distribution may result from the diffuse engraftment of human immune cells in the humanized mouse model. While this differs from the region-specific vulnerability often seen in HAND patients, the model still captures clinically relevant features of neuroHIV, including neuroinflammation and partial treatment responsiveness. The hallmarks of HIV-1 infection in humans (e.g., peripheral viral load and human CD4+ T-cell decline) are reflected in these humanized mice. In addition to our current data, metabolic encephalopathy induced by viral infection resulted in micro- and astro-gliosis, excitotoxicity, myelin injury, and neuronal injury, as seen previously in both humans and infected mice [63, 82]. Such a spectrum of pathologies makes the humanized mice well-suited for studying HIV-1-induced neuropathology and treatment effects, despite inherent differences from human HAND.

This study represents the first application of CEST MRI to neuroHIV, serving as a preliminary feasibility investigation to monitor plausible neurometabolic biomarkers of HAND pathology [27, 83] and their changes following ART. The observed changes in CEST contrasts associated with glutamate, creatine, and NOE-linked macromolecules demonstrate the ability of CEST MRI to image HIV-linked neurocognitive impairment. Similarly, CEST detection of contrast restoration represents a novel technique for evaluating ART effectiveness non-invasively. The neurometabolic biomarkers identified herein could serve as potential tools for early detection and longitudinal monitoring of HAND.

Future research should address the limitations of the current study, including the restricted sample size, the absence of behavioral evaluation, and the lack of long-term follow-up. In addition, the lack of an uninfected (HIV−) control group is a notable limitation. Because this was an exploratory feasibility study focused on longitudinal infection- and ART-related changes, all available animals were allocated to the infected±ART groups, and baseline (Week 0) scans were used as each animal’s internal reference. Future studies will incorporate dedicated HIV-control mice scanned in parallel to more clearly distinguish infection-specific effects from age- or procedure-related changes. Furthermore, although we performed IHC for viral and glial markers, we were unable to perform quantitative or metabolite-specific IHC due to limited tissue availability and the lack of region-matched serial sections. As a result, GFAP- and Iba-1–based assessments of glial activation were necessarily qualitative. We now clarify this explicitly. Future studies will address this limitation by collecting dedicated, anatomically consistent tissue series for region-specific, quantitative IHC and more direct correlation analyses between imaging-derived contrasts and immunohistological markers. Compared to conventional magnetic resonance spectroscopy (MRS), CEST MRI can offer improved spatial resolution and sensitivity to specific molecular exchanges under optimized conditions. However, CEST relies on indirect detection, making it susceptible to confounding factors such as B_0_ and B_1_ inhomogeneities, pH effects, and overlapping exchangeable proton pools, which can limit its specificity. MRS, in contrast, directly quantifies metabolite concentrations with higher biochemical specificity, albeit at lower spatial resolution. Integrating CEST with complementary modalities such as MRS and diffusion tensor imaging (DTI) will be instrumental in improving mechanistic interpretability and validating CEST-based biomarkers. Furthermore, exploring adjunctive therapies targeting residual macromolecular alterations may provide deeper insights into HAND pathogenesis and therapeutic strategies.

## Conclusions

This study emphasizes the promising potential of CEST MRI in elucidating HIV-1-associated neurometabolic alterations in a humanized mouse model. Through the innovative application of CEST MRI, this research highlights ART’s neuroprotective effects on the neural metabolic profile while revealing persistent macromolecular changes, such as those potentially linked to membrane lipids. These findings underscore the valuable role of MRI as a non-invasive tool for detecting and monitoring HAND-related changes, offering new insights into both metabolic disruptions and therapeutic outcomes. Collectively, these results support the broader adoption of MRI in neuroHIV research to enhance diagnostic accuracy, therapeutic efficacy, and overall management of HAND pathogenesis. However, given the exploratory nature of this work, further longitudinal and multimodal studies will be essential to validate CEST-based biomarkers and clarify their translational relevance. Ultimately, advancing MRI-based biomarkers may help improve long-term neurological health and quality of life for PLWH.

## Supplementary Material

Suppl. Material

**Supplementary Material:** This article contains supplementary material (https://doi.org/10.1515/nipt-2025-0017).

## Data Availability

The raw data can be obtained on request from the corresponding author.

## References

[1] Panel on Antiretroviral Guidelines for Adults and Adolescents. Guidelines for the use of antiretroviral agents in adults and adolescents with HIV. In: Health and Human Services. Rockville, MD: Office of AIDS Research, National Institutes of Health; 2023.

[2] Kemnic TR, Gulick PG. HIV antiretroviral therapy. In: StatPearls [Internet]. Treasure Island (FL): StatPearls Publishing; 2025. Available from: https://www.ncbi.nlm.nih.gov/books/NBK513308/ [Accessed 20 Sep 2022].

[3] Cohn LB, Chomont N, Deeks SG. The biology of the HIV-1 latent reservoir and implications for cure strategies. Cell Host Microbe 2020;27:519–30. 32272077 10.1016/j.chom.2020.03.014 PMC7219958

[4] Gaebler C, Nogueira L, Stoffel E, Oliveira TY, Breton G, Millard KG, Prolonged viral suppression with anti-HIV-1 antibody therapy. Nature 2022;606:368–74. 35418681 10.1038/s41586-022-04597-1 PMC9177424

[5] Ndung’u T, McCune JM, Deeks SG. Why and where an HIV cure is needed and how it might be achieved. Nature 2019;576:397–405. 31853080 10.1038/s41586-019-1841-8 PMC8052635

[6] Pasternak AO, Berkhout B. HIV persistence: silence or resistance? Curr Opin Virol 2023;59:101301. 36805974 10.1016/j.coviro.2023.101301

[7] Saylor D, Dickens AM, Sacktor N, Haughey N, Slusher B, Pletnikov M, HIV-associated neurocognitive disorder–pathogenesis and prospects for treatment. Nat Rev Neurol 2016;12:234–48. 26965674 10.1038/nrneurol.2016.27 PMC4937456

[8] Eggers C, Arendt G, Hahn K, Husstedt IW, Maschke M, Neuen-Jacob E, HIV-1-associated neurocognitive disorder: epidemiology, pathogenesis, diagnosis, and treatment. J Neurol 2017;264:1715–27. 28567537 10.1007/s00415-017-8503-2 PMC5533849

[9] Kompella S, Al-Khateeb T, Riaz OA, Orimaye SO, Sodeke PO, Awujoola AO, HIV-Associated neurocognitive disorder (HAND): relative risk factors. Curr Top Behav Neurosci 2021;50:401–26. 32720161 10.1007/7854_2020_131

[10] Campbell LM, Fennema-Notestine C, Saloner R, Hussain M, Chen A, Franklin D, Use of neuroimaging to inform optimal neurocognitive criteria for detecting HIV-Associated brain abnormalities. J Int Neuropsychol Soc 2020;26:147–62. 31576785 10.1017/S1355617719000985 PMC7015796

[11] Chaganti J, Brew BJ. MR spectroscopy in HIV associated neurocognitive disorder in the era of cART: a review. AIDS Res Ther 2021;18:65. 34625091 10.1186/s12981-021-00388-2 PMC8501619

[12] Chaganti J, Marripudi K, Staub LP, Rae CD, Gates TM, Moffat KJ, Imaging correlates of the blood-brain barrier disruption in HIV-associated neurocognitive disorder and therapeutic implications. AIDS 2019;33:1843–52. 31274535 10.1097/QAD.0000000000002300

[13] McLaurin KA, Booze RM, Mactutus CF. Diagnostic and prognostic biomarkers for HAND. J Neurovirol 2019;25:686–701. 30607890 10.1007/s13365-018-0705-6 PMC6610810

[14] Sreeram S, Ye F, Garcia-Mesa Y, Nguyen K, El Sayed A, Leskov K, The potential role of HIV-1 latency in promoting neuroinflammation and HIV-1-associated neurocognitive disorder. Trends Immunol 2022;43:630–9. 35840529 10.1016/j.it.2022.06.003 PMC9339484

[15] Wang W, Liu D, Wang Y, Li R, Liu J, Liu M, Frequency-dependent functional alterations in people living with HIV with early stage of HIV-associated neurocognitive disorder. Front Neurosci 2022;16:985213. 36699529 10.3389/fnins.2022.985213 PMC9868721

[16] Olivier IS, Cacabelos R, Naidoo V. Risk factors and pathogenesis of HIV-associated neurocognitive disorder: the role of host genetics. Int J Mol Sci 2018;19. 10.3390/ijms19113594. PMC6274730 30441796

[17] Al-Ghananeem AM, Smith M, Coronel ML, Tran H. Advances in brain targeting and drug delivery of anti-HIV therapeutic agents. Expet Opin Drug Deliv 2013;10:973–85. 10.1517/17425247.2013.781999 23510097

[18] Caruana G, Vidili G, Serra PA, Bagella P, Spanu A, Fiore V, The burden of HIV-associated neurocognitive disorder (HAND) in post-HAART era: a multidisciplinary review of the literature. Eur Rev Med Pharmacol Sci 2017;21:2290–301. 28537651

[19] Destache CJ. Chapter 12- brain as an HIV sequestered site: use of nanoparticles as a therapeutic option. Prog Brain Res 2009;180:225–33. 20302837 10.1016/S0079-6123(08)80012-X

[20] Gonzalez H, Podany A, Al-Harthi L, Wallace J. The far-reaching hand of cART: cART effects on astrocytes. J Neuroimmune Pharmacol 2021;16:144–58. 32147775 10.1007/s11481-020-09907-w PMC7483784

[21] Rao KS, Ghorpade A, Labhasetwar V. Targeting anti-HIV drugs to the CNS. Expet Opin Drug Deliv 2009;6:771–84. 10.1517/17425240903081705 PMC2754315 19566446

[22] Zhang YL, Ouyang YB, Liu LG, Chen DX. Blood-brain barrier and neuro-AIDS. Eur Rev Med Pharmacol Sci 2015;19:4927–39. 26744885

[23] Gannon P, Khan MZ, Kolson DL. Current understanding of HIV-associated neurocognitive disorders pathogenesis. Curr Opin Neurol 2011;24:275–83. 21467932 10.1097/WCO.0b013e32834695fb PMC3683661

[24] Gill AJ, Kolson DL. Chronic inflammation and the role for cofactors (Hepatitis C, drug abuse, antiretroviral drug toxicity, aging) in hand persistence. Curr HIV AIDS Rep 2014;11:325–35. 24929842 10.1007/s11904-014-0210-3 PMC4188391

[25] Gray LR, Roche M, Flynn JK, Wesselingh SL, Gorry PR, Churchill MJ. Is the central nervous system a reservoir of HIV-1? Curr Opin HIV AIDS 2014;9:552–8. 25203642 10.1097/COH.0000000000000108 PMC4215931

[26] Longino AA, Paul R, Wang Y, Lama JR, Brandes P, Ruiz E, HIV disease dynamics and markers of inflammation and CNS injury during primary HIV infection and their relationship to cognitive performance. J Acquir Immune Defic Syndr 2022;89:183–90. 34629415 10.1097/QAI.0000000000002832 PMC8752485

[27] Ernst T, Jiang CS, Nakama H, Buchthal S, Chang L. Lower brain glutamate is associated with cognitive deficits in HIV patients: a new mechanism for HIV-associated neurocognitive disorder. J Magn Reson Imag 2010;32:1045–53. 10.1002/jmri.22366 PMC2967445 21031507

[28] Sailasuta N, Shriner K, Ross B. Evidence of reduced glutamate in the frontal lobe of HIV-seropositive patients. NMR Biomed 2009;22:326–31. 18988228 10.1002/nbm.1329

[29] Wang Z, Pekarskaya O, Bencheikh M, Chao W, Gelbard HA, Ghorpade A, Reduced expression of glutamate transporter EAAT2 and impaired glutamate transport in human primary astrocytes exposed to HIV-1 or gp120. Virology 2003;312: 60–73. 12890621 10.1016/s0042-6822(03)00181-8

[30] Porcheray F, Léone C, Samah B, Rimaniol AC, Dereuddre-Bosquet N, Gras G. Glutamate metabolism in HIV-infected macrophages: implications for the CNS. Am J Physiol Cell Physiol 2006;291:C618–26. 16687472 10.1152/ajpcell.00021.2006

[31] Vázquez-Santiago FJ, Noel RJ Jr., Porter JT, Rivera-Amill V. Glutamate metabolism and HIV-associated neurocognitive disorders. J Neurovirol 2014;20:315–31. 24867611 10.1007/s13365-014-0258-2 PMC4098898

[32] Sailasuta N, Ross W, Ananworanich J, Chalermchai T, DeGruttola V, Lerdlum S, Change in brain magnetic resonance spectroscopy after treatment during acute HIV infection. PLoS One 2012;7:e49272. 23229129 10.1371/journal.pone.0049272 PMC3500278

[33] Dahmani S, Kaliss N, VanMeter JW, Moore DJ, Ellis RJ, Jiang X. Alterations of brain metabolites in adults with HIV: a systematic meta-analysis of magnetic resonance spectroscopy studies. Neurology 2021;97:e1085–96. 34253633 10.1212/WNL.0000000000012394 PMC8456358

[34] Thirion A, Loots DT, Williams ME, Solomons R, Mason S. (1)H-NMR metabolomics investigation of CSF from children with HIV reveals altered neuroenergetics due to persistent immune activation. Front Neurosci 2024;18:1270041. 38745940 10.3389/fnins.2024.1270041 PMC11091326

[35] Deme P, Rojas C, Slusher BS, Rais R, Afghah Z, Geiger JD, Bioenergetic adaptations to HIV infection. Could modulation of energy substrate utilization improve brain health in people living with HIV-1? Exp Neurol 2020;327:113181. 31930991 10.1016/j.expneurol.2020.113181 PMC7233457

[36] Doke M, McLaughlin JP, Cai JJ, Pendyala G, Kashanchi F, Khan MA, HIV-1 Tat and cocaine impact astrocytic energy reservoirs and epigenetic regulation by influencing the LINC01133-hsa-miR-4726–5p-NDUFA9 axis. Mol Ther Nucleic Acids 2022;29:243–58. 35892093 10.1016/j.omtn.2022.07.001 PMC9307901

[37] Liu G, Song X, Chan KW, McMahon MT. Nuts and bolts of chemical exchange saturation transfer MRI. NMR Biomed 2013;26:810–28. 23303716 10.1002/nbm.2899 PMC4144273

[38] van Zijl PC, Yadav NN. Chemical exchange saturation transfer (CEST): what is in a name and what isn’t? Magn Reson Med 2011;65:927–48. 21337419 10.1002/mrm.22761 PMC3148076

[39] Ward KM, Aletras AH, Balaban RS. A new class of contrast agents for MRI based on proton chemical exchange dependent saturation transfer (CEST). J Magn Reson 2000;143:79–87. 10698648 10.1006/jmre.1999.1956

[40] Wu B, Warnock G, Zaiss M, Lin C, Chen M, Zhou Z, An overview of CEST MRI for non-MR physicists. EJNMMI Phys 2016;3:19. 27562024 10.1186/s40658-016-0155-2 PMC4999387

[41] Cember ATJ, Nanga RPR, Reddy R. Glutamate-weighted CEST (gluCEST) imaging for mapping neurometabolism: an update on the state of the art and emerging findings from in vivo applications. NMR Biomed 2023;36:e4780. 35642353 10.1002/nbm.4780

[42] Orzyłowska A, Oakden W. Saturation transfer MRI for detection of metabolic and microstructural impairments underlying neurodegeneration in Alzheimer’s disease. Brain Sci 2021;12. 10.3390/brainsci12010053. PMC8773856 35053797

[43] Chung J, Sun D, Hitchens TK, Modo M, Bandos A, Mettenburg J, Dual contrast CEST MRI for pH-weighted imaging in stroke. Magn Reson Med 2024;91:357–67. 37798945 10.1002/mrm.29842 PMC10872804

[44] Cui J, Afzal A, Zu Z. Comparative evaluation of polynomial and Lorentzian lineshape-fitted amine CEST imaging in acute ischemic stroke. Magn Reson Med 2022;87:837–49. 34590729 10.1002/mrm.29030 PMC9293005

[45] Heo HY, Tee YK, Harston G, Leigh R, Chappell MA. Amide proton transfer imaging in stroke. NMR Biomed 2023;36:e4734. 35322482 10.1002/nbm.4734 PMC9761584

[46] Kim H, Krishnamurthy LC, Sun PZ. Brain pH imaging and its applications. Neuroscience 2021;474:51–62. 33493621 10.1016/j.neuroscience.2021.01.026

[47] Bagga P, Pickup S, Crescenzi R, Martinez D, Borthakur A, D’Aquilla K, In vivo GluCEST MRI: reproducibility, background contribution and source of glutamate changes in the MPTP model of Parkinson’s disease. Sci Rep 2018;8:2883. 29440753 10.1038/s41598-018-21035-3 PMC5811435

[48] Debnath A, Hariharan H, Nanga RPR, Reddy R, Singh A. Glutamate-weighted CEST contrast after removal of magnetization transfer effect in human brain and rat brain with tumor. Mol Imaging Biol 2020;22:1087–101. 31907844 10.1007/s11307-019-01465-9

[49] Hadar PN, Kini LG, Nanga RPR, Shinohara RT, Chen SH, Shah P, Volumetric glutamate imaging (GluCEST) using 7T MRI can lateralize nonlesional temporal lobe epilepsy: a preliminary study. Brain and Behavior 2021;11:e02134. 34255437 10.1002/brb3.2134 PMC8413808

[50] Jia Y, Chen Y, Geng K, Cheng Y, Li Y, Qiu J, Glutamate chemical exchange saturation transfer (GluCEST) magnetic resonance imaging in pre-clinical and clinical applications for encephalitis. Front Neurosci 2020;14:750. 32848546 10.3389/fnins.2020.00750 PMC7399024

[51] Li R, Dai Z, Hu D, Zeng H, Fang Z, Zhuang Z, Mapping the alterations of glutamate using Glu-weighted CEST MRI in a rat model of fatigue. Front Neurol 2020;11. 10.3389/fneur.2020.589128. PMC7673456 33250853

[52] Neal A, Moffat BA, Stein JM, Nanga RPR, Desmond P, Shinohara RT, Glutamate weighted imaging contrast in gliomas with 7 tesla magnetic resonance imaging. Neuroimage: Clinic 2019;22:101694. 10.1016/j.nicl.2019.101694 PMC6396013 30822716

[53] Zhuang Z, Shen Z, Chen Y, Dai Z, Zhang X, Mao Y, Mapping the changes of glutamate using glutamate chemical exchange saturation transfer (GluCEST) technique in a traumatic brain injury model: a longitudinal pilot study. ACS Chem Neurosci 2019;10:649–57. 30346712 10.1021/acschemneuro.8b00482

[54] Cai K, Singh A, Poptani H, Li W, Yang S, Lu Y, CEST signal at 2ppm (CEST@2ppm) from Z-spectral fitting correlates with creatine distribution in brain tumor. NMR Biomed 2015;28:1–8. 25295758 10.1002/nbm.3216 PMC4257884

[55] Chen L, Wei Z, Cai S, Li Y, Liu G, Lu H, High-resolution creatine mapping of mouse brain at 11.7 T using non-steady-state chemical exchange saturation transfer. NMR Biomed 2019;32:e4168. 31461196 10.1002/nbm.4168

[56] Chen L, Zeng H, Xu X, Yadav NN, Cai S, Puts NA, Investigation of the contribution of total creatine to the CEST Z-spectrum of brain using a knockout mouse model. NMR Biomed 2017;30. 10.1002/nbm.3834. PMC5685917 28961344

[57] Khlebnikov V, van der Kemp WJM, Hoogduin H, Klomp DWJ, Prompers JJ. Analysis of chemical exchange saturation transfer contributions from brain metabolites to the Z-spectra at various field strengths and pH. Sci Rep 2019;9:1089. 30705355 10.1038/s41598-018-37295-y PMC6355971

[58] Lee JS, Xia D, Jerschow A, Regatte RR. In vitro study of endogenous CEST agents at 3 T and 7 T. Contrast Media Mol Imaging 2016;11:4–14. 26153196 10.1002/cmmi.1652 PMC4706513

[59] Singh A, Debnath A, Cai K, Bagga P, Haris M, Hariharan H, Evaluating the feasibility of creatine-weighted CEST MRI in human brain at 7 T using a Z-spectral fitting approach. NMR Biomed 2019;32:e4176. 31608510 10.1002/nbm.4176 PMC11463199

[60] Goerke S, Soehngen Y, Deshmane A, Zaiss M, Breitling J, Boyd PS, Relaxation-compensated APT and rNOE CEST-MRI of human brain tumors at 3 T. Magn Reson Med 2019;82:622–32. 30927313 10.1002/mrm.27751

[61] Jones CK, Huang A, Fau - Xu J, Xu J, Fau - Edden RAE, Edden, Nuclear overhauser enhancement (NOE) imaging in the human brain at 7T. (1095–9572 (Electronic)). 10.1016/j.neuroimage.2013.03.047 PMC3848060 23567889

[62] Zhao Y, Sun C, Zu Z. Assignment of molecular origins of NOE signal at −3.5 ppm in the brain. Magn Reson Med 2023;90:673–85. 36929814 10.1002/mrm.29643 PMC10644915

[63] Bade AN, Gorantla S, Dash PK, Makarov E, Sajja BR, Poluektova LY, Manganese-enhanced magnetic resonance imaging reflects brain pathology during progressive HIV-1 infection of humanized mice. Mol Neurobiol 2016;53:3286–97. 26063593 10.1007/s12035-015-9258-3 PMC4676748

[64] Boska MD, Dash PK, Knibbe J, Epstein AA, Akhter SP, Fields N, Associations between brain microstructures, metabolites, and cognitive deficits during chronic HIV-1 infection of humanized mice. Mol Neurodegener 2014;9:58. 25523827 10.1186/1750-1326-9-58 PMC4297430

[65] Nair AB, Jacob S. A simple practice guide for dose conversion between animals and human. J Basic Clin Pharm 2016;7:27–31. 27057123 10.4103/0976-0105.177703 PMC4804402

[66] Keene CM, Griesel R, Zhao Y, Gcwabe Z, Sayed K, Hill A, Virologic efficacy of tenofovir, lamivudine and dolutegravir as second-line antiretroviral therapy in adults failing a tenofovir-based first-line regimen. AIDS 2021;35:1423–32. 33973876 10.1097/QAD.0000000000002936 PMC7612028

[67] Dorward J, Drain PK, Garrett N. Point-of-care viral load testing and differentiated HIV care. Lancet HIV 2018;5:e8–9. 29290227 10.1016/S2352-3018(17)30211-4 PMC6003416

[68] Hill A, Clayden P, Thorne C, Christie R, Zash R. Safety and pharmacokinetics of dolutegravir in HIV-positive pregnant women: a systematic review. J Virus Erad 2018;4:66–71. 29682297 10.1016/S2055-6640(20)30247-8 PMC5892677

[69] The LH. End resistance to dolutegravir roll-out. Lancet HIV 2020;7:e593. 32890495 10.1016/S2352-3018(20)30231-9

[70] Foster EG, Sillman B, Liu Y, Summerlin M, Kumar V, Sajja BR, Long-acting dolutegravir formulations prevent neurodevelopmental impairments in a mouse model. Front Pharmacol 2023;14. 10.3389/fphar.2023.1294579. PMC10750158 38149054

[71] Zhang C, Su H, Waight E, Poluektova LY, Gorantla S, Gendelman HE, Accelerated neuroimmune dysfunction in aged HIV-1-Infected humanized mice. Pharmaceuticals 2024;17. 10.3390/ph17020149. PMC10892358 38399364

[72] Su H, Cheng Y, Sravanam S, Mathews S, Gorantla S, Poluektova LY, Immune activations and viral tissue compartmentalization during progressive HIV-1 infection of humanized mice. Front Immunol 2019;10:340. 30873181 10.3389/fimmu.2019.00340 PMC6403174

[73] Dash PK, Kaminski R, Bella R, Su H, Mathews S, Ahooyi TM, Sequential LASER ART and CRISPR treatments eliminate HIV-1 in a subset of infected humanized mice. Nat Commun 2019;10:2753. 31266936 10.1038/s41467-019-10366-y PMC6606613

[74] Kim M, Gillen J, Landman BA, Zhou J, van Zijl PC. Water saturation shift referencing (WASSR) for chemical exchange saturation transfer (CEST) experiments. Magn Reson Med 2009;61:1441–50. 19358232 10.1002/mrm.21873 PMC2860191

[75] Bade AN, Gendelman HE, McMillan J, Liu Y. Chemical exchange saturation transfer for detection of antiretroviral drugs in brain tissue. AIDS 2021;35:1733–41. 34049358 10.1097/QAD.0000000000002960 PMC8373768

[76] Liu Y, Gauthier GC, Gendelman HE, Bade AN. Dual-peak lorentzian CEST MRI for antiretroviral drug brain distribution. NeuroImmune Pharm Ther 2023;2:63–9. 37027345 10.1515/nipt-2022-0012 PMC10070014

[77] Jacobs PS, Jee J, Fang L, Devlin E, Iannelli C, Thakuri D, Application of glutamate weighted CEST in brain imaging of nicotine dependent participants in vivo at 7T. PLoS One 2024;19:e0297310. 38363747 10.1371/journal.pone.0297310 PMC10871471

[78] Cai Z, Zhong Q, Feng Y, Wang Q, Zhang Z, Wei C, Non-invasive mapping of brown adipose tissue activity with magnetic resonance imaging. Nat Metab 2024;6:1367–79. 39054361 10.1038/s42255-024-01082-z PMC11272596

[79] Wu Q, Qi Y, Gong P, Huang B, Cheng G, Liang D, Fast and robust pulsed chemical exchange saturation transfer (CEST) MRI using a quasi-steady-state (QUASS) algorithm at 3 T. Magn Reson Imaging 2024;105:29–36. 37898416 10.1016/j.mri.2023.10.009

[80] Dickens AM, Anthony DC, Deutsch R, Mielke MM, Claridge TD, Grant I, Cerebrospinal fluid metabolomics implicate bioenergetic adaptation as a neural mechanism regulating shifts in cognitive states of HIV-infected patients. AIDS 2015;29:559–69. 25611149 10.1097/QAD.0000000000000580 PMC4340743

[81] Avgerinos KI, Spyrou N, Bougioukas KI, Kapogiannis D. Effects of creatine supplementation on cognitive function of healthy individuals: a systematic review of randomized controlled trials. Exp Gerontol 2018;108:166–73. 29704637 10.1016/j.exger.2018.04.013 PMC6093191

[82] Bade AN, Zhou B, Epstein AA, Gorantla S, Poluektova LY, Luo J, Improved visualization of neuronal injury following glial activation by manganese enhanced MRI. J Neuroimmune Pharmacol 2013;8:1027–36. 23729245 10.1007/s11481-013-9475-3 PMC3746563

[83] Keller MA, Venkatraman TN, Thomas A, Deveikis A, LoPresti C, Hayes J, Altered neurometabolite development in HIV-infected children: correlation with neuropsychological tests. Neurology 2004;62:1810–7. 15159483 10.1212/01.wnl.0000125492.57419.25

