# Supplementary material for: CEST MRI assessment of HIV-1-associated neurometabolic impairments in a humanized mouse model
Source: NeuroImmune Pharm Ther. Author manuscript; Available in PMC 2026 Feb 20. (PMC12920017; doi:10.1515/nipt-2025-0017)
Supplement: Suppl. Material [file NIHMS2144153-supplement-Suppl__Material.docx]

**B**

**A**

**D**

**C**


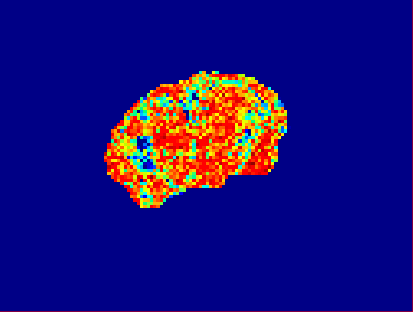

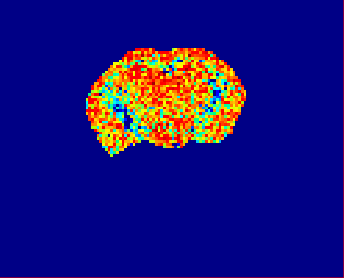

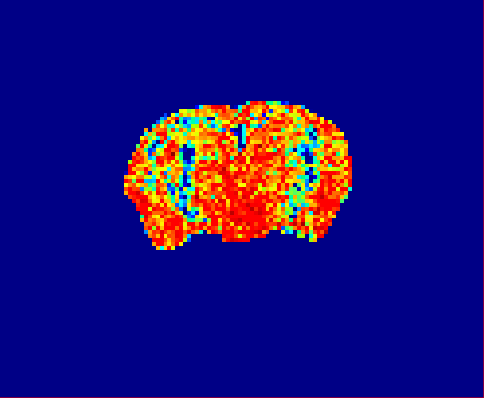

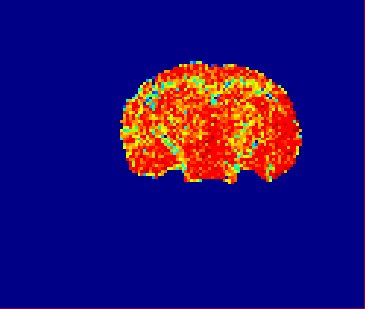

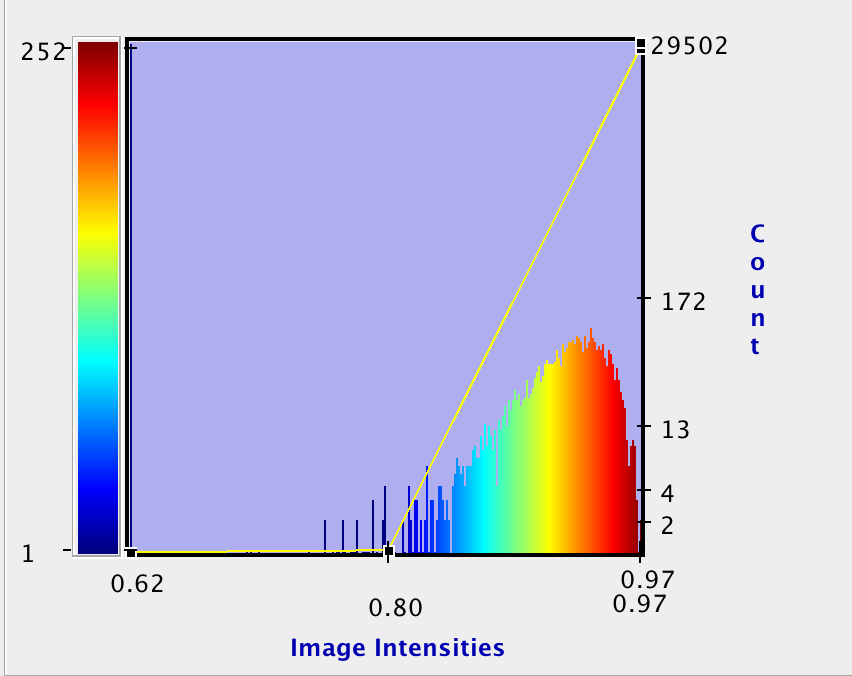


0.8

1.0

Baseline

HIV (6 WPI)

HIV (12 WPI)

ART (12 WPI)

**E**

**Supplemental Figure 2** Representative Z-spectra, 5-pool Lorentzian fitting curves, and residuals from mice in each experimental group: (**A**) Baseline, (**B**) HIV (6 WPI), (**C**) HIV (12 WPI), and (**D**) ART (12 WPI). Z-spectra were averaged over all brain voxels. (**E**) R² heatmaps of representative mice from each group, visualizing the voxel-wise goodness-of-fit. R² (coefficient of determination) reflects the quality of the Lorentzian model fit

**A**

**B**

**C**

**Supplemental Figure 3.** Normality assessment of CEST contrasts using quantile–quantile (Q–Q) plots for each brain region: (**A**) 3 ppm (glutamate-associated) CEST contrast, (**B**) 2 ppm (creatine-associated) CEST contrast, and (**C**) NOE at −3.5 ppm. The data distributions in all regions demonstrated strong adherence to normality assumptions.

↓ 2 ppm CEST (creatine-related signal)

↓ 3 ppm CEST (glutamate-related signal)

↔ NOE (macromolecules)

0-6 WPI

6-12 WPI

Vehicle

↓ 2 ppm CEST (creatine-related signal)

↑ NOE (macromolecules)

↑ 2 ppm CEST (creatine-related signal)

↑ 3 ppm CEST (glutamate-related signal)

↔ NOE (macromolecules)

6-12 WPI

ART

**Supplemental Figure 4.** Schematic illustration summarizing the progression of neurometabolic changes detected by CEST MRI across infection stages. An early decline in the 2 ppm creatine-related signal (0–6 WPI) may reflect initial astrocytic energy stress. By 12 WPI, reductions in the 3 ppm glutamate-related signal may indicate impaired glutamate-glutamine cycling and disrupted neuron-glia metabolic coupling. Both 2 ppm and 3 ppm contrasts showed recovery following ART. In contrast, elevations in NOE at –3.5 ppm appear early and persist through 12 WPI, potentially reflecting macromolecular or membrane-related alterations that recover more slowly than metabolite-based measures.

**Supplemental Table 1.** Maximum, minimum, and median R² values (coefficient of determination) across all brain voxels for each experimental group. These values reflect the overall quality of Lorentzian fitting in CEST data processing.

|  | Baseline | HIV (6 WPI) | HIV (12 WPI) | ART (12 WPI) |
| --- | --- | --- | --- | --- |
| Max R^2^ | 0.993 | 0.993 | 0.993 | 0.992 |
| Min R^2^ | 0.988 | 0.980 | 0.975 | 0.990 |
| Median R^2^ | 0.991 | 0.990 | 0.988 | 0.990 |
